# Dissecting the Complexity of Skeletal-Malocclusion-Associated Phenotypes: Mouse for the Rescue

**DOI:** 10.3390/ijms24032570

**Published:** 2023-01-29

**Authors:** Iqbal M. Lone, Osayd Zohud, Aysar Nashef, Christian Kirschneck, Peter Proff, Nezar Watted, Fuad A. Iraqi

**Affiliations:** 1 Department of Clinical Microbiology and Immunology, Sackler Faculty of Medicine, Tel-Aviv University, Tel-Aviv 6997801, Israel; 2 Department of Oral and Maxillofacial Surgery, Baruch Padeh Medical Center Poriya, Poriya 1520800, Israel; 3 Department of Orthodontics, University Hospital of Regensburg, University of Regensburg, 93047 Regensburg, Germany; 4 Center for Dentistry Research and Aesthetics, Jatt 4491800, Israel; 5 Department of Orthodontics, Faculty of Dentistry, Arab America University, Jenin P.O. Box 240, Palestine; 6 Gathering for Prosperity Initiative, Jatt 4491800, Israel

**Keywords:** skeletal-malocclusion, complexity, genetic analysis, gene mapping, GWAS, EWAS, gene expression

## Abstract

Skeletal deformities and malocclusions being heterogeneous traits, affect populations worldwide, resulting in compromised esthetics and function and reduced quality of life. Skeletal Class III prevalence is the least common of all angle malocclusion classes, with a frequency of 7.2%, while Class II prevalence is approximately 27% on average, varying in different countries and between ethnic groups. Orthodontic malocclusions and skeletal deformities have multiple etiologies, often affected and underlined by environmental, genetic and social aspects. Here, we have conducted a comprehensive search throughout the published data until the time of writing this review for already reported quantitative trait loci (QTL) and genes associated with the development of skeletal deformation-associated phenotypes in different mouse models. Our search has found 72 significant QTL associated with the size of the mandible, the character, shape, centroid size and facial shape in mouse models. We propose that using the collaborative cross (CC), a highly diverse mouse reference genetic population, may offer a novel venue for identifying genetic factors as a cause for skeletal deformations, which may help to better understand Class III malocclusion-associated phenotype development in mice, which can be subsequently translated to humans. We suggest that by performing a genome-wide association study (GWAS), an epigenetics-wide association study (EWAS), RNAseq analysis, integrating GWAS and expression quantitative trait loci (eQTL), micro and small RNA, and long noncoding RNA analysis in tissues associated with skeletal deformation and Class III malocclusion characterization/phenotypes, including mandibular basic bone, gum, and jaw, in the CC mouse population, we expect to better identify genetic factors and better understand the development of this disease.

## 1. Introduction

The intricate morphology of the facial skeleton plays a variety of important tasks. It provides crucial anatomy for oral communication, eating, and facial expression as well as housing and protecting the majority of sensory organs and the forebrain. Variation in the biological processes that regulate facial growth is what causes structured differences (covariance) in facial shape and between facial form and size [1,2]. Examining the genotype–phenotype (GP) map at various levels is necessary to comprehend how these mechanisms function and interact to form the facial skeleton [3].

Skeletal patterns and malocclusions are heterogeneous conditions affecting populations worldwide, resulting in compromised esthetics and speech function and reduced quality of life [4]. The World Health Organization (WHO) considers malocclusion to be among the most important oral health problems after caries and periodontitis [5]. Its prevalence varies widely and is estimated at 39% to 93% in children and adolescents, respectively [6,7]. This prevalence has a very wide spectrum and clinical heterogeneity. The inhomogeneity may be due to the ethnic and age differences among the patients considered in studies evaluating the prevalence of malocclusion [8,9]. Class III prevalence is the least common of all angle malocclusion classes, with a mean frequency of 7.2%.

The prevalence of class III malocclusion varies between different populations and races. Prevalence among Asians is high, while it is low among Caucasians [10,11,12,13,14]. Nonetheless, in the Middle East, the prevalence of class III malocclusion is considered high compared to Caucasians, but relatively low compared to Far East Asians [13,14]. The prevalence of Class III among Saudis has been reported as 9.4% [15]. The most complex and difficult orthodontic problem to diagnose and treat is class III malocclusion [16]. The prevalence of this type of malocclusion ranges from 0.2% in the white population to almost 12% in the Chinese and Japanese population and up to 20% in the Saudi population [17,18].

The purpose of this report is to evaluate genetic associations between candidate craniofacial genes and skeletal variations in patients with skeletal malocclusions in mouse models. This study characterizes phenotypes of the craniofacial skeleton in mice with malocclusion and generates genetic data on craniofacial genes/loci to identify phenotype-genotype interactions. Several previous research results suggested genetic pathways that play a role in the anteroposterior and vertical skeletal variation observed in patients with malocclusion and in the type of skeletal malocclusion. Future studies should also consider examining soft-tissue differences to uncover the genetic etiology of skeletal and soft-tissue variations in patients with malocclusion. Identifying the genetic influence on these phenotypes will improve our understanding of the molecular regulation of postnatal facial growth and inform clinical practitioners to improve treatment efficacy for patients with malocclusion. Furthermore, for a better understanding of the complexity and heterogeneity of human class III malocclusion, developing a new class III malocclusion mouse model would suffice. To determine the effects of genetic variation on class III malocclusion signaling networks and phenotypic variability, we reviewed all the reported studies in the mouse model for investigating the genetic basis of mandibular size and prognathism, which we believe will help improve the understanding the human skeletal Class III malocclusion.

## 2. Genetics of Skeletal Class III Malocclusion Phenotype

In recent years, it has been widely recognized that both genetic and environmental factors contribute to class III malocclusion development [19,20]. Various loci and suggested genes associated with class III malocclusion, linkage analysis and association studies were identified [20,21]. There is a strong association between mandibular prognathism and genetics that is well documented through studies conducted primarily with family members and twin siblings. The expression of the phenotype is a product of genetics and environmental factors. The multifactorial nature of mandibular prognathism makes it difficult to study and understand. Mandibular prognathism has a prevalence of only 1% in white people but up to 15% in Asian populations [21,22].

Identifying these genetic factors and understanding their mechanisms would aid in the diagnosis, prediagnosing, predicting and treatments. Single-nucleotide polymorphisms (SNPs) and the haplotypes defined by shared SNPs can be genotyped to determine normal and variable craniofacial phenotypes [23]. Studies have shown that some SNPs (P561T, C422F and I526L) occuring in the growth hormone receptor (GHR) gene are associated with mandibular ramus height in Japanese, Korean, and Chinese populations and the P561T polymorphism has an inhibitory effect on mandibular growth in young children [20,24,25,26,27]. Multiple SNPs have been found to be involved in mandibular prognathism [22,28,29]. A SNP of the noggin gene (rs1348322) was present in four families with mandibular micrognathia [30].

## 3. Genome-Wide Association Study (GWAS)

Recently, five genome-wide nonparametric linkage analyses and one genome-wide association study (GWAS) for identifying susceptibility loci associated with mandibular prognathism and skeletal Class III phenotype showed conflicting results in humans [19,20,21]. However, inconsistent genes within the exome sequence for mandibular prognathism have been demonstrated. The differences in genotype for the same phenotype (mandibular prognathism) could be due to the different breeds and the definition of mandibular prognathism (different degrees of the deficient size of the maxilla and mandible).

## 4. Epigenetics and Skeletal Class III Malocclusion

The epigenetic regulation is a reversible, heritable change in chromatin structures that control genes’ turning on and off. The proposed epigenetic mechanisms are via DNA methylation, RNA interference, histone modifications, or a combination of these processes [31]. In eukaryotic nucleus cells, genomic DNA is assembled in the histone protein nucleus (H2A, H2B, H3, and H4), which forms nucleosomes, which are then condensed into chromatin fibers [32]. Histone modification in masseter muscle genes by acetylation can increase gene expression of type IIX (fast) MHC by opening the chromatin region, making it more loose and more accessible to transcription complexes [33]. On the other hand, histone deacetylation causes closure of chromatin, which reduces type I (slow twitch) MHC, since methylated cytosines make it inaccessible for RNA polymerase to undergo a transcription process [34]. Overall, from the sagittal dimension perspective, histone deacetylase 4 (HDAC4) and lysine acetyltransferase 6B (KAT6B) gene expression was increased in the masseter muscle of patients with class III malocclusion compared to class II malocclusion [33,35]. KAT6B activates RUNX2, which encodes the osteogenic transcription factor. Epigenetic control of KAT6B and HDAC4 coordination is important in the development of malocclusion and musculoskeletal complexes. Epigenetic studies of class III malocclusion showed that HDAC4 seemed to associate favorably with rapid myosin genes, particularly type IIX, and adversely with slow myosin type I [35].

## 5. Mouse Model for Studying Mandibular Size and Prognathism

Controlled and standardized investigations of the genetics of complex human diseases are challenging because of the cumulative effect and interactions of numerous genes and environmental factors. Despite major discoveries of genetic risk factors for some diseases, the specific genes involved in susceptibility to most complex diseases and the mechanisms translating genetic effects into disease susceptibility are commonly unknown. The mouse model is a powerful tool for dissecting the genetic bases of complex phenotypes/diseases (multifactorial phenotypes). Once the genes associated and underlined a given phenotype are identified in the mouse, these results subsequently can be translated to humans using comparative and orthologue analysis.

In a previous study by Dohmoto et al. [36], it was found that in mice, mandibular size is controlled by genes located on chromosomes 10 and 11, corresponding to human chromosome regions 12q21 and 2p13, respectively. If orthodontists could clinically predict whether patients have strong factors for mandibular overgrowth, they would be able to select strategies to treat mandibular prognathism more effectively. If a growing orthodontic patient with mandibular prognathism has genetic factors such as variants in candidate genes, orthodontists could opt for orthodontic surgery instead of long-term use of a chincap appliance for the patient [36].

## 6. Quantitative Trait Loci Analysis in Mouse Model

Mandibular morphology analysis has long been used for tribe identification by discriminant analysis [37]. In such studies, 11 to 13 measurement points placed on the outline of the mandible, used to characterize mandibular shape and statistical analysis is performed to identify the strain or subline [38]. However, the discriminant analysis was not extended to include a genetic analysis. Previous research has suggested that the effects of genes on the mandible should be spatially patterned [39]. Signaling interactions coordinate the outgrowth of the facial rudiments from buds of undifferentiated mesenchyme into the intricate array of bone and cartilage structures that, along with muscle and other tissues, make up the adult face [40]. The relationship between phenotypes and the genes responsible for mandibular shape is difficult to uncover, since polygenes are involved in facial development, including mandibular growth. Quantitative trait loci (QTL) analysis has been very successful in identifying chromosomal regions, with quantitative effects dependent on polygenes such as body weight and susceptibility to alcoholism [41,42]. Recombinant inbred (RI) strains of mice are valuable tools for studying complex traits such as body weight [43]. RI strains are derived from systematic inbreeding of randomly selected F2-generation pairs of a cross between two different inbred mouse strains.

Dohmoto et al. [36] focused on identifying the chromosomal regions involved in the regulation of the anteroposterior length of the mandible as indicated by the distance between the sites corresponding to the menton and the gonion. The report on genetic analysis of mandibular size (length between the menton and gonion) in SMXA RI strain using QTL analysis showed that a suggestive QTL on chromosome 10, and two significant QTL were detected in males and females. In the proximal region of chromosome 11, two significant QTL have been identified in females. These QTL could prove crucial for studying class III malocclusion and developing strategies for its prevention [36].

The SM/J allele on chromosome 10 for QTL is associated with a large lower jaw. The size of the mandible was not only determined by genes located in chromosomes 10 and 11, as there were also some effects that were weak in other chromosomes. The Mouse Genome Database (http://www.informatics.jax.org/ accessed on 25 January 2023) was searched for candidate genes according to their position at approximately 60cM of chromosome 10 and between 13cM and 16cM of chromosome 11, identifying more than 10 genes as candidates for the mandibular size revealed in chromosomes 10 and 11. It is interesting that a candidate gene near the QTL for mandibular size on chromosome 11 is Otx1 (orthodentices), a gene closely related to Otx2. Mouse embryos homozygous for a knockout allele of Otx2 display a striking phenotype, in which the entire brain is absent rostrally to rhombomere 3 [44]. This clearly shows the importance of this gene for the development of the rostral head. The Otx1 knockout mice display a less severe phenotype, but nonetheless indicate a crucial role for Otx1 in vertebrate head development [45]. Based on these studies, we suggest that Otx1 is a potential candidate gene that might be involved in the development of mandibular size.

The positions around 60cM of mouse chromosome 10 and between 13cM and 16cM of mouse chromosome 11 correspond to orthologue regions 12q21 and 2p13, respectively, in human chromosomes. If the result of this study is to be transferred to clinical diagnoses, the focus can be placed on the two chromosome regions 12q21 and 2p13. It may be possible to predict the size of a patient’s mandible before the growth of the maxillofacial bones is complete by looking for the polymorphisms of these chromosomal regions, whether they come from large or small mandibles. However, additional analysis involving fine-scale mapping will be necessary to elucidate the precise nature of the QTL. Here, in this paper, we have summarized in Table 1, the currently mapped QTL, their locations, and confidence intervals (CI) of significant mandibular phenotypes in mice.

## 7. Mouse Knockout Gene

Fontoura et al. [47] assessed the associations between facial skeletal variations and the type of skeletal malocclusion using 71 candidate craniofacial genes/loci in patients with malocclusion. Two genes, SNAI3 and TWIST1, proved to be particularly relevant for craniofacial variation after Bonferroni correction for multiple testing. Although statistically insignificant after Bonferroni correction, SNPs within FGFR2, EDN1, TBX5, and COL1A1 showed suggestive associations with the type of skeletal malocclusion [47].

It is possible that the phenotypes represented in PC1 and PC4 are more complex in their genetic etiology than those captured in PC2 and PC3 and are associated with a greater number of genetic variants, each with a small effect. If this is the case, large-sample GWAS may be a more effective approach to identify loci for such phenotypes, since there is little evidence that they are associated with the candidate genes and loci evaluated. The main results suggest that SNAI3 is associated with craniofacial variations from highly concave to convex profiles, while TWIST1 is related to variations from short to long mandibular bodies. SNAI3 is a member of the SNAIL family of zinc finger transcription factors important at epithelial to mesenchymal junctions that contribute to the formation of the mesoderm and neural crest [48]. Snai3 is expressed in the facial prominences that give rise to the upper and lower jaws (EMAGE http://www.emouseatlas.org/emage/home.php accessed on 25 January 2023). Interestingly, Snai3 null mice show no significant abnormalities, suggesting that Snail genes are complementary in their biological functions [49]. Snai2 and Snai3 double knockout mice exhibit abnormalities beyond the Snai2−/−null, including ocular deformities and growth failure [49]. The neural crest-specific deletion of Snai1 on a Snai2−/− background results in multiple craniofacial defects including mandibular deficiency similar to the Pierre Robin sequence [50], indicating that SNAIL genes can modulate jaw growth [50]. Therefore, future studies of this gene family and its role in malocclusion are warranted. TWIST1 encodes a basal helix-loop-helix transcription factor that plays an important role in activating downstream mesodermal genes during early development in Drosophila [51]. Mutations and deletions in human TWIST1 are found in patients with the Saethre-Chotzen syndrome [52], a condition associated with a wide spectrum of craniofacial abnormalities including craniosynostosis, maxillary hypoplasia, narrow palate, facial asymmetry with a deviated nasal septum and cleft palate [53]. Twist1+/mice show craniosynostosis mainly affecting the coronal sutures [54]. Given that maxillary hypoplasia is a common finding in patients with craniosynostosis, one might speculate that genetic variation in TWIST1 may also result in premature ossification of the maxillary sutures, resulting in a class III malocclusion due to maxillary hypoplasia. In addition, Twist1 is necessary for mandibular ossification, cusp development, and molar development [55]. Given the evidence for genetic interactions between TWIST and members of the SNAI family during mesoderm development, the phenotypic relationships we found in both SNAI3 and TWIST1 are quite intriguing [51] and in cranial suture ossification [56]. Whether TWIST and SNAIL act independently or interactively to increase the risk of abnormal maxillomandibular relationships in malocclusion warrants future research with a larger sample.

## 8. The Collaborative Cross (CC) Mouse Population as a Powerful Tool for Systematic Genetic Analysis of Class III Malocclusion

Many limitations in studies of human populations can be addressed in mice, which share human susceptibility to many pathogens and environmental circumstances. Once the genes associated and underlining a given trait are identified in the mouse, they subsequently can be identified in humans using comparative and orthologue analysis. However, standard laboratory mouse lines have low genetic diversity and thus limited usefulness for the study of genetic variation in complex traits. To remedy this, the Collaborative Cross (CC), a new genetically highly diverse set of recombinant inbred mouse strains, was designed. With a focus on features important to human health, the collaborative cross CC strains were created as a new tool for high-resolution mapping and identification of the genetic elements underlying complex phenotypes. The creation of the mouse CC genetic reference population (GRP) was motivated by the need to model genetic variety. The wide panel of recombinant inbred (RI) strains that make up this special GRP resource were specifically developed for complicated trait analysis [57,58,59,60] and suggest a stronger power than any previously published method [61,62]. This model was developed by mating three wild-derived strains (CAST/Ei, derived from M. m. castaneum mice, PWK/PhJ, and WSB/EiJ, derived from wild *M. musculus* and wild *M. domesticus* mice, respectively) and five classical inbred lines (A/J, C57BL/6J, 129S1/SvImJ, NOD/LtJ, and NZO/HiLt). The final population of CC mice has a significant genetic variety due in part to this phylogenetic variation. The map growth in the CC population is around four times greater, which increases the proportionate accuracy of the QTL map position. Since all genetic traits are homozygous due to their inbred origin, each QTL’s genetic variance is increased. Additionally, environmental sources of variance can be reduced by phenotyping numerous individuals in each line. When compared to typical F2 mapping populations, the set of RILs’ effective mapping power is multiplied many times over in this way.

**Figure 1:**
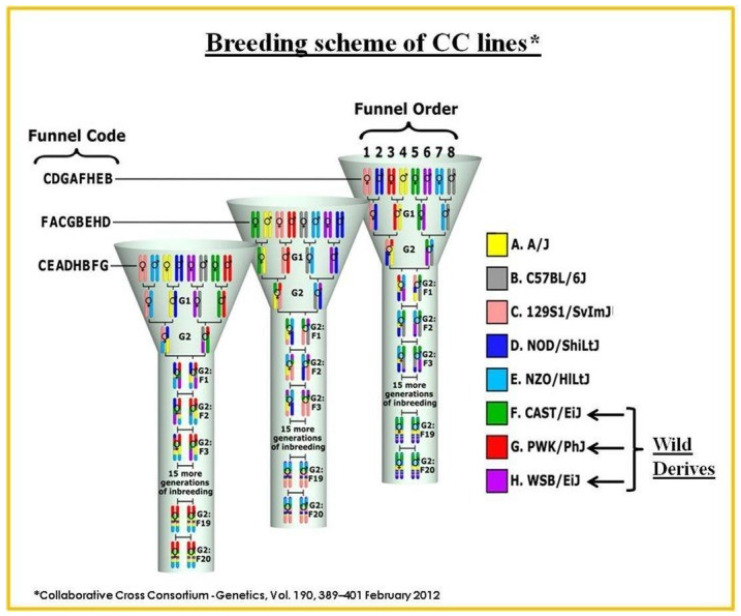
The development of the Collaborative Cross (CC) mouse model. This breeding approach was designed to randomize the genetic composition of each inbred line. A single breeding funnel results in a recombinant CC inbred line representing the genomes of the eight founders of CC mice. The eight founding tribes are arranged in different positions. Each CC line originates from an independently breeding funnel of eight CC founders so that at every recombination site in the CC population is uniquely generated.

The genomes of the eight CC founder strains are introduced into a single CC lineage with a well-planned breeding schedule. After more than 20 generations of brother-sister mating, following the introduction of genomes from the parental founding strains at the G2-F1 stage, it was expected that approximately 99% homozygosity would be achieved with an approximately equivalent contribution from the eight founding strains [57,58,59,60,61]. This GRP has higher genetic variations (over 36 million SNPs) than what is present in the natural human population, which has approximately 20 million SNPs, greater when compared to other mouse inbred lines (4.4 million SNPS), and relatively high level of recombination events (separation between founders) [58]. A recent stimulation study on QTL analysis using the CC population showed that the resolution of the imaged interval could be within less than 1 Mb [57,62,63,64]. CC strains offer a unique opportunity for performing GWAS and mapping quantitative trait loci (QTL) and identify candidate genes associated with mandibular size and prognathism, as a basis for class III malocclusion spontaneous malocclusion phenotype, while reducing the environmental stress. It is strongly believed that the high genetic diversity of the founding strains of CC mice provides a strong basis for identifying novel genetic loci associated with these defined phenotypes and subsequently proceed with validation by mouse knockout gene, conditional knockout, CRISPER-Cas9. The single breeding funnel resulting in a recombinant CC inbred line representing the genomes of the eight founders of CC mice is represented in Figure 1.

## 9. Future Direction for Comprehensively Dissecting the Complexity of Skeletal Class III Malocclusion Genes in CC Mice and in Humans

A new class III malocclusion model should be created, taking into consideration the diversity of the human population, in order to better understand the hereditary elements that may be implicated in the beginning, progression, and establishment of skeletal class III malocclusion. The first thorough review of the molecular basis of complex features has been provided by systems genetic analysis, which is helpful for pinpointing the genes, signaling pathways, and networks that underlie prevalent disorders. In order to examine the associations of various skeletal Class III malocclusion phenotypes, data on cellular, molecular, and clinical aspects are then merged. The regulatory genomic areas are implicated in phenotypic variance in both in vitro and in vivo monitored traits when SNP genotype data from each CC lineage are combined. The potential for parallel in vitro/in vivo screening in such an experimental design, as well as the advancements in high-throughput assessment technologies and computational techniques, may help us comprehend how the interaction of various genetic alterations affects the onset and severity of class III malocclusion. Then, human systems can be used to apply verified gene-gene interactions and/or gene-environment networks to inform risk for class III malocclusion prevention or pharmaceutical targets. Systems genetics will most likely be able to comprehend both the biology and the severity of the disease by comprehending the mechanisms of the genetic loci (QTL and genes) identified in the genome-wide association study (GWAS) that contribute to susceptibility to skeletal class III malocclusion diseases.

Previously, it was shown that integrating GWAS and expression quantitative trait loci (eQTL) data is a powerful analytical framework to predict potential susceptibility genes for complex diseases [65]. Therefore, we highly expect that including this approach in the study of skeletal class III malocclusion will enhance the understanding of the development of this disease.

Regulatory RNAs are now extensively investigated on the molecular basis regarding various diseases. Still, to our knowledge, hardly any study is available regarding the status of these molecules in skeletal class III malocclusion. Here, we suggest that these regulatory RNAs will be of pivotal importance studying this condition and will lead to a better understanding of the molecular basis of this disease.

**Figure 2:**
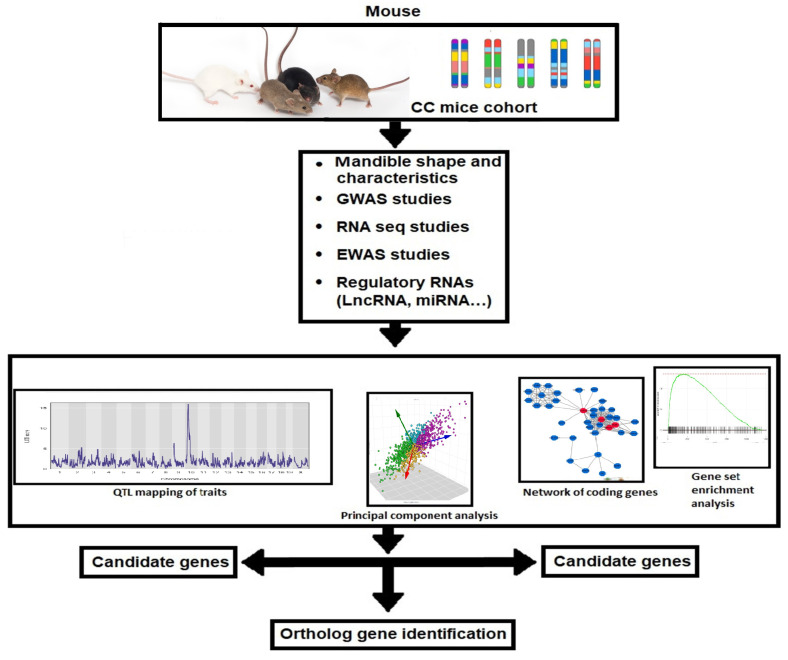
Workflow for the creation of system genetic databases from the CC population, which is incredibly heterogeneous and may have vastly different susceptibilities to class III malocclusion. Class II and III malocclusion in mice is checked. The analysis of numerous associations between malocclusion and class III traits uses cellular, molecular, and clinical trait data. The regulatory genomic areas are implicated in phenotypic variance in both in vitro and in vivo monitored traits when SNP genotype data from each CC lineage are combined. Combining data with later candidate gene association research in humans may help find susceptibility genes linked to the emergence of class III malocclusion in people.

The workflow diagram for the generation of system genetic datasets from the highly diverse CC population and their susceptibility to developing skeletal class III malocclusion is represented in Figure 2. The diagram shows the two-way integrations of the two suggested venues for studying Skeletal Class III malocclusion, which will increase the power for dissecting this complex disease.

## 10. Conclusions

It is thought that the genetic influence on skeletal class II and III malocclusion susceptibility cannot be fully understood using only methods intended to pinpoint the primary impacts of specific genotypes/alleles in humans. To better understand the complexity and heterogeneity of human class II and III malocclusion, it is important to develop a new class II and III malocclusion mouse model. The proposal to perform a systems genetic analysis using CC GRP mice to characterize the impact of genetic variation on class II and III malocclusion signaling networks and phenotypic diversity is a promising starting point.

## Figures and Tables

**Table 1:** Locations and confidence intervals (CI) of significant QTLs for the size (mean of the two sides) of the mandible characters (M), shape (SH), centroid size (C) and facial shape principal components (PCs) given as map distances from the nearest proximal marker and from the centromere after QTL analysis in mouse models.

Chr	QTL	C.I. (cM)	Reference
1	QTL-SH1.1	38–62	[39]
	QTL-SH1.2	78–120	[39]
	QTL-M1.1	12–38	[46]
	QTL-M1.2	49–65	[46]
	PC1	52–53	[1]
2	QTL-SH2.1	—	[39]
	QTL-C2.1	—	[39]
	QTL-M2.1	21–40	[46]
	QTL-M2.2	62–70	[46]
3	QTL-SH3.1	37–53	[39]
	QTL-C3.1	27–61	[39]
	QTL-M3.1	14–24	[46]
	QTL-M3.2	69–75	[46]
4	QTL-SH4.1	28–46	[39]
	QTL-C4.1	18–42	[39]
	QTL-M4.1	40–60	[46]
5	QTL-SH5.1	37–105	[39]
	QTL-CU5.1	26–62	[39]
	QTL-M5.1	29–70	[46]
6	QTL-SH6.1	4–16	[39]
	QTL-SH6.2	74–98	[39]
	QTL-C6.1	70–100	[39]
	QTL-M6.1	3–19	[46]
	PC1	99–100.2	[1]
	PC2	3.5–6	[1]
7	QTL-SH7.1	13–65	[39]
	QTL-SH7.2	61–77	[39]
	QTL-C7.1	1–85	[39]
	QTL-M7.1	37–75	[46]
	PC1	58–66	[1]
8	QTL-M8.1	16–36	[46]
9	QTL-SH9.1	16–38	[39]
	QTL-SH9.2	54–88	[39]
	QTL-M9.1	41–65	[46]
10	QTL-SH10.1	9–41	[39]
	QTL-SH10.2	65–75	[39]
	QTL-C10.1	63–87	[39]
	QTL-CS10.1	55–83	[39]
	QTL-M10.1	16–28	[46]
	QTL-M10.2	31–57	[46]
11	QTL-SH11.1	13–31	[39]
	QTL-SH11.2	63–97	[39]
	QTL-C11.1	17–55	[39]
	QTL-C11.2	67–109	[39]
	QTL-CS11.1	51–109	[39]
	QTL-M11.1	4–47	[46]
12	QTL-SH12.1	21–33	[39]
	QTL-SH12.2	29–70	[39]
	QTL-SU12.1	43–63	[39]
	QTL-M12.1	32–49	[46]
	PC3	8.5–17	[1]
13	QTL-SH13.1	79–97	[39]
	QTL-C13.1	7–45	[39]
	QTL-M13.1	37–59	[46]
14	QTL-SH14.1	48–64	[39]
	QTL-C14.1	24–64	[39]
	QTL-M14.1	5–27	[46]
	QTL-M14.2	38–48	[46]
	PC2	22–24	[1]
15	QTL-SH15.1	29–53	[39]
	QTL-SH15.2	53–85	[39]
	QTL-C15.1	41–67	[39]
	QTL-SS15.1	11–55	[39]
	QTL-M15.1	27–43	[46]
16	QTL-SH16.1	14–44	[39]
	QTL-M16.1	15–33	[46]
17	QTL-C17.1	11–19	[39]
	QTL-M17.1	29–45	[46]
	PC4	45–48	[1]
18	QTL-SH18.1	61–91	[39]
	QTL-M18.1	25–73	[46]
19	QTL-SH19.1	15–61	[39]
